# A Case Report of an Isolated Cardiac Metastasis in a Patient with Esophageal Carcinoma

**DOI:** 10.7759/cureus.44717

**Published:** 2023-09-05

**Authors:** Rem Aziz, Tina Hsu, Hadi Toeg, Sudhir R Sundaresan, Kristopher Dennis

**Affiliations:** 1 Medicine, University of British Columbia, Vancouver, CAN; 2 Medical Oncology, The Ottawa Hospital, University of Ottawa, Ottawa, CAN; 3 Cardiac Surgery, University of Ottawa Heart Institute, Ottawa, CAN; 4 Thoracic Surgery, University of Ottawa, Ottawa, CAN; 5 Radiation Oncology, The Ottawa Hospital, University of Ottawa, Ottawa, CAN

**Keywords:** palliative care, cardiac surgery, radiotherapy, chemotherapy, cardiac metastasis, esophageal cancer

## Abstract

A 76-year-old Caucasian male presented with syncope, intermittent melena, anemia, and unexplained weight loss. Esophagogastroduodenoscopy revealed a friable non-obstructing esophageal tumor that appeared thickened on computed tomography (CT). Biopsies confirmed a poorly differentiated carcinoma. Fluorine-18 fluorodeoxyglucose positron emission tomography/CT (F-18 FDG PET/CT) showed intense FDG avidity with a maximum standardized uptake value (SUV_max_) of 23. Although CT did not identify any lymphadenopathy or distant metastases, a mildly enhancing lobulated circumscribed mass with no internal calcification was incidentally identified in the left atrium. Cardiac magnetic resonance imaging (MRI) favored myxoma over thrombus given the signal characteristics and mild enhancement; however, F-18 FDG PET/CT showed an SUV_max_ of 18, more consistent with a metastasis. The cardiac mass was resected and shown to be a metastatic focus of poorly differentiated carcinoma, histologically identical to the esophageal mass. He received a single 8 Gray (Gy) fraction of urgent hemostatic radiotherapy for his primary tumor followed by palliative chemotherapy with cisplatin, capecitabine, and pembrolizumab. He was readmitted for transfusion due to recurrent bleeding from his primary tumor and given a second urgent hemostatic fraction of 8 Gy for stabilization. Systemic therapy was eventually discontinued due to declining performance status. He received consolidative palliative radiotherapy (20Gy in five fractions) but continued to deteriorate over the next three months and died in hospice, ten months from the time of his initial presentation.

## Introduction

Cardiac metastases are rare compared to metastases to other sites, although the malignant spread of primary tumors can indeed affect the epicardium, myocardium, endocardium, great vessels, coronary arteries, and lymphatic network [1]. The incidence of cardiac metastases is difficult to ascertain and likely underestimated as most are discovered in post-mortem studies [1,2]. It primarily occurs in the sixth and seventh decades of life, with no apparent gender predilection [2]. Reported cases have become more common over the past 40 years, likely due to improved diagnostic technologies and management plans that have extended the survival and life expectancy of affected patients [3,4].

Most cardiac metastases originate from primary lung cancers, followed by breast cancers, hematologic malignancies, and melanomas [5]. This reflects the high prevalence and aggressive nature of these tumors whereas in contrast, prostate cancer, although more prevalent in men than any of the above tumors, rarely metastasizes to the heart. Other tumors including ovarian, gastric, renal, and pancreatic carcinomas have also been associated with cardiac metastasis [1,4,6].

Solitary cardiac metastases are extremely rare; most cardiac metastases are found in patients with disseminated disease and poor expected prognoses with limited therapeutic options [1,2]. Clinical manifestations are variable and dependent on the anatomic cardiac sub-site and degree of invasion. While most patients are asymptomatic at presentation, others may have pericardial effusion, tamponade, valvular dysfunction, heart failure, ventricular or supraventricular heart rhythm disturbances, conduction defects, syncope, embolism, or other non-specific symptoms [2]. Direct extension from primary tumors and regional lymphatics is the most frequent route for cardiac invasion, whereas hematogenous spread to the myocardium is thought to be less common [1].

Practice patterns vary and depend on patient, disease, and available treatment factors; there is no gold standard of care. Patients are treated with chemotherapy, radiotherapy, or a combination of both, with surgical resection usually being reserved for cases of limited metastatic disease of a single lesion [7].

Our article aims to contribute to the limited available literature by describing the case of a 76-year-old Caucasian male with an isolated cardiac metastasis secondary to poorly differentiated esophageal carcinoma.

## Case presentation

A 76-year-old Caucasian male presented with syncope and disclosed a several weeks’ history of intermittent melena-type stools and an unexplained 75-pound weight loss in the preceding six months. His presenting hemoglobin was 73 g/L. He had a past medical history of smoking, hypertension, vasectomy, pericarditis, and a small melanoma treated 15 years earlier with excision but no adjuvant therapy.

**Figure 1:**
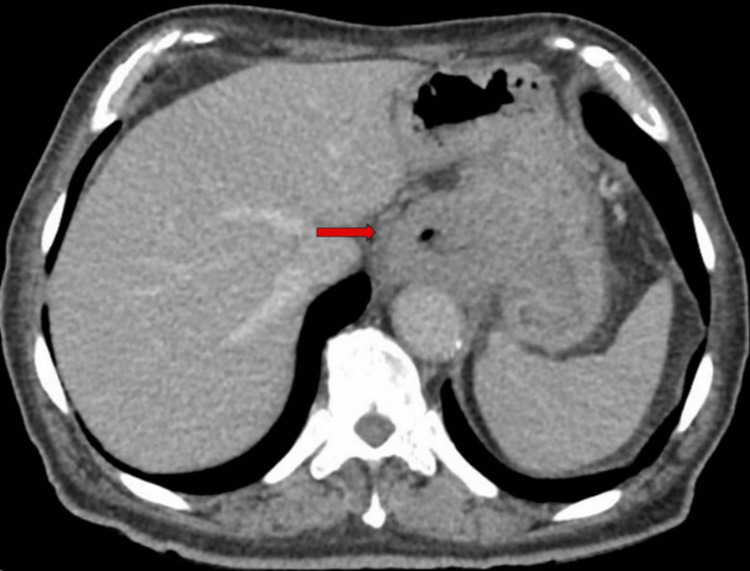
CT image showing primary tumor in the axial plane (red arrow). CT, computed tomography

**Figure 2:**
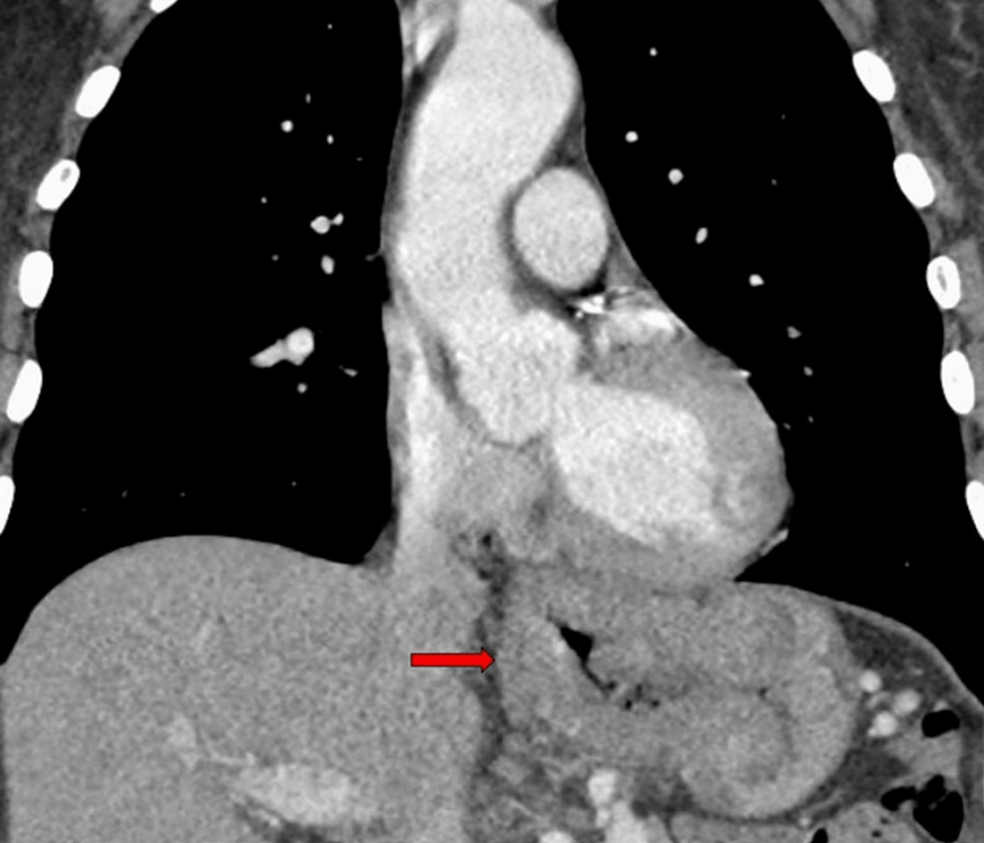
CT image showing primary tumor in coronal plane (red arrow). CT, computed tomography

Esophagogastroduodenoscopy revealed a friable non-obstructing tumor extending 30-48 cm from the incisors, spanning the lower esophagus, esophagogastric junction, and gastric fundus. The endoscopic ultrasound (EUS) probe was not tolerated. Biopsies of the esophageal and gastric aspects of the mass collectively confirmed a poorly differentiated carcinoma; overall, markers to distinguish squamous vs. glandular differentiation were scant or negative and unhelpful in distinguishing between squamous carcinoma or adenocarcinoma. Neoplastic cells stained positive for pan-cytokeratin had scant staining with CK7 and were negative for p40, CK-5, Napsin, CD45, and Sox10. There was no loss of nuclear expression of mismatch repair (MMR) proteins. Computed tomography (CT) of the head, chest, abdomen, and pelvis showed thickening of the esophageal and gastric walls at the site of the tumor seen on endoscopy (Figure 1 and Figure 2) but no convincing lymphadenopathy or distant metastases.

**Figure 3:**
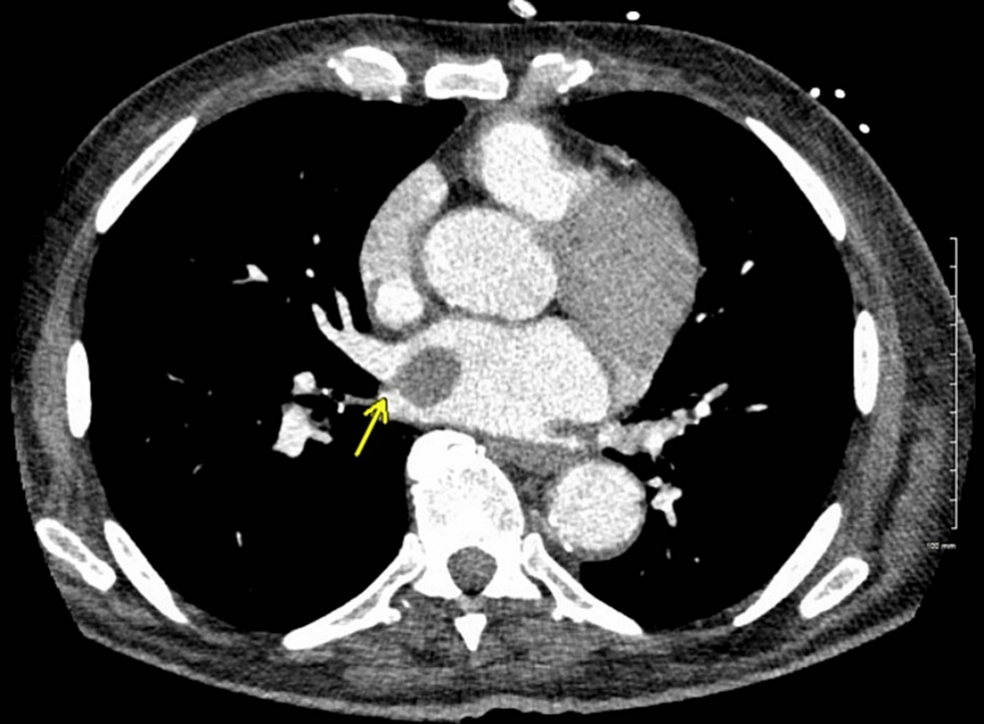
CT image showing lobulated circumscribed mass (yellow arrow) attached to the right lateral wall of the left atrium. CT, computed tomography

**Figure 4:**
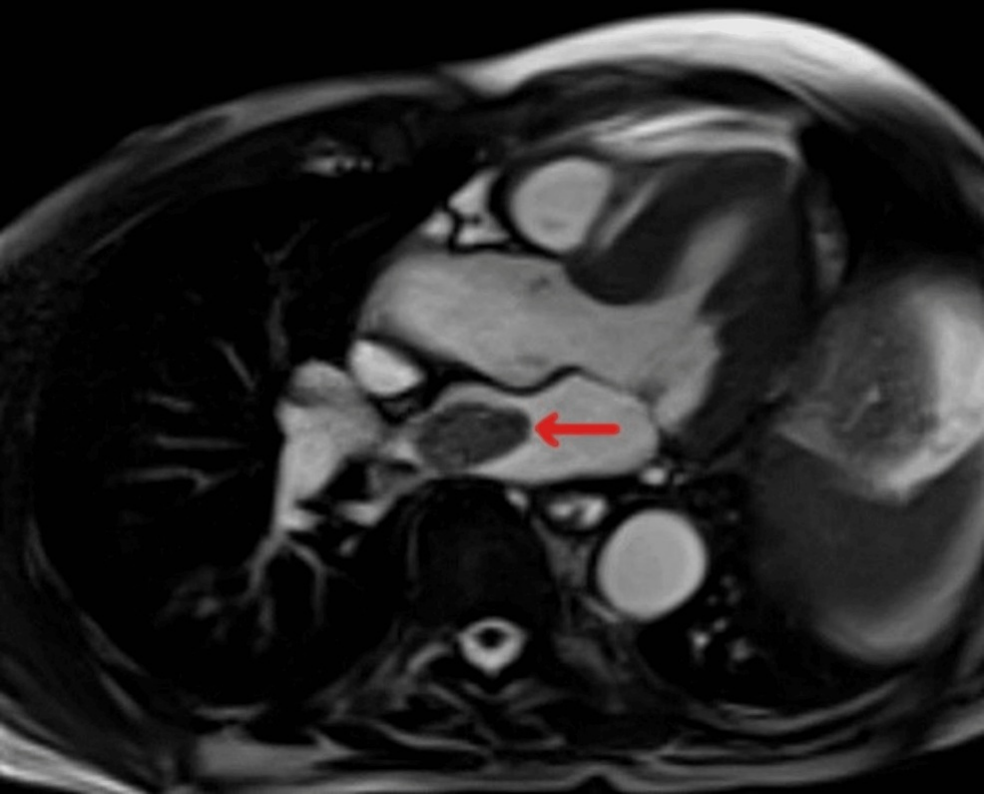
Cardiac MRI showing left atrial mass on 4C CINES image (red arrow). MRI, magnetic resonance imaging

**Figure 5:**
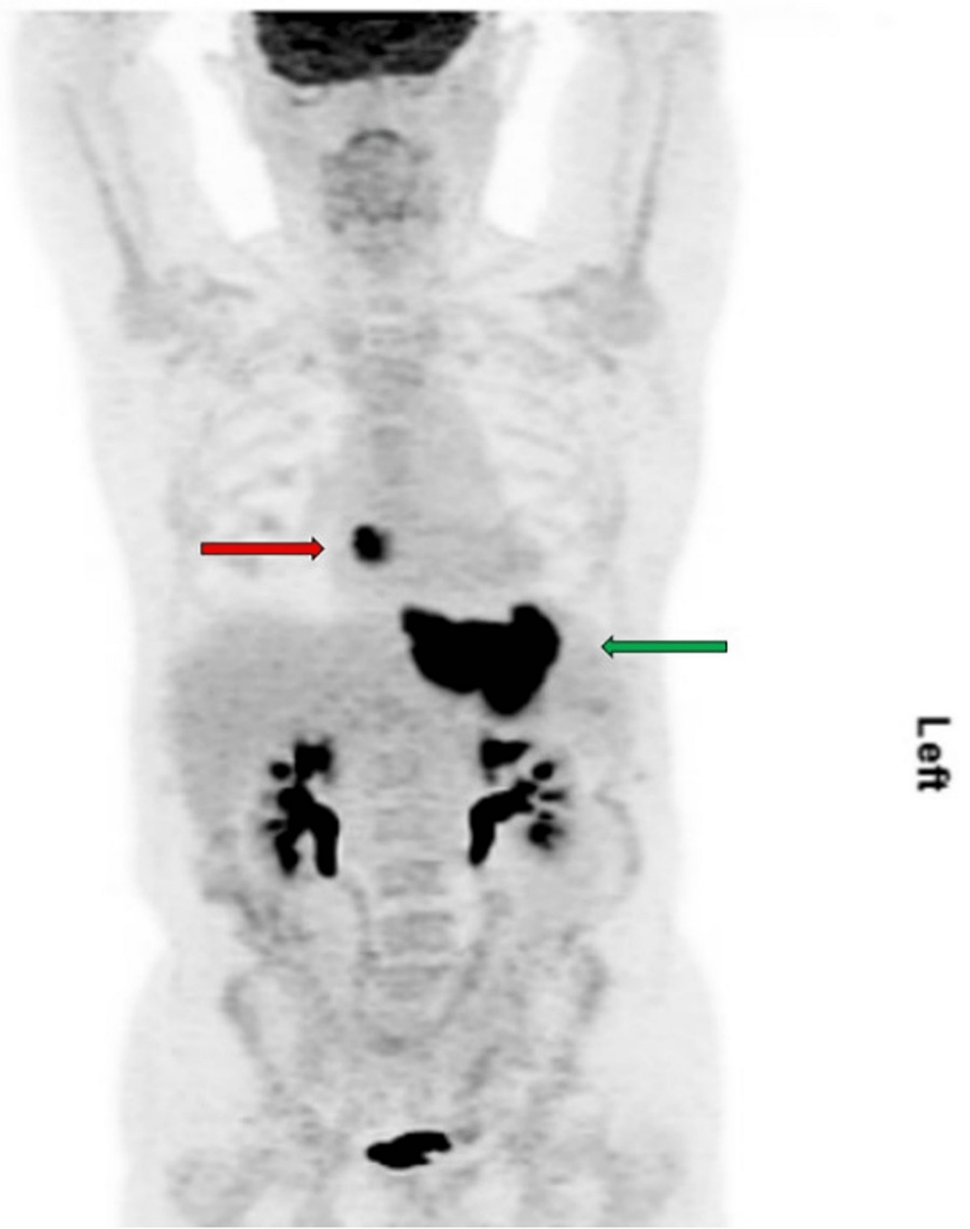
F18-FDG PET image showing left atrial mass (red arrow) and primary tumor (green arrow). F18-FDG PET, fluorine-18 fluorodeoxyglucose positron emission tomography

Incidentally, the scan identified a 2.9×2.0×2.6 cm mildly enhancing lobulated circumscribed mass with no internal calcifications attached to the right lateral wall of the left atrium by a pedicle (Figure 3). The differential diagnosis included myxoma and thrombus. Cardiac magnetic resonance imaging (MRI) measured the mass at 3.0×1.8 cm. It was T1 hypointense (Figure 4) and T2/SPAIR hyperintense with minimal delayed enhancement. Myxoma was favored over thrombus given the signal characteristics and mild enhancement. Fluorine-18 fluorodeoxyglucose positron emission tomography/CT (F-18 FDG PET/CT) showed intense FDG avidity at the esophagogastric mass with a maximum standardized uptake value (SUV_max_) of 23. The left atrial mass also showed intense avidity with an SUV_max_ of 18, more consistent with metastasis than a myxoma or thrombus (Figure 5). There was no lymphadenopathy or other potential distant metastases.

He received a single 8 Gray (Gy) fraction of urgent hemostatic radiotherapy to his primary tumor and stabilized hemodynamically. For both diagnostic and therapeutic reasons, he underwent resection of the atrial mass via median sternotomy. Intraoperatively, the mass appeared smooth and multicolored with a small component of the thrombus. It was attached to the confluence of the right upper and lower pulmonary veins and was removed in its entirety. Pathological examination confirmed the mass to be a poorly differentiated carcinoma, histologically identical to the esophageal mass.

Although his only site of metastatic disease had been resected, it was not clear if he would ultimately undergo surgical resection of the primary tumor. Thus, he was treated with palliative intent chemotherapy with pembrolizumab (200 mg day 1), cisplatin (80 mg/m^2^ day 1), and capecitabine (825 mg/m^2^×14 days), every 21 days. It was hoped that after a period of disease stability, he might be considered for more aggressive local therapies. Cisplatin was switched to carboplatin (AUC 4) after three cycles due to acute kidney injury.

He otherwise had incidental bilateral pulmonary emboli identified on CT and began low molecular weight heparin. He was admitted shortly afterward due to recurrent bleeding from his primary tumor with a hemoglobin of 55 g/L, for which he was transfused. Anticoagulation was held, and a second urgent hemostatic fraction of 8Gy was delivered to stabilize him hemodynamically.

At six months following the initial presentation, repeat F-18 FDG PET/CT showed unchanged disease status at the primary tumor site. However, due to persistent fatigue, poor dietary intake, and a tenuous performance status, systemic therapy was discontinued and he was deemed an inoperable candidate.

He received 20 Gy in five fractions of consolidative palliative radiotherapy, with target delineation aided by platinum fiducial markers that were injected into mucosal blebs at the cranial and caudal tumor borders under EUS guidance. In subsequent weeks, his overall performance status slowly declined, but he was devoid of pain and bleeding and could eat solid foods without dysphagia. He died in hospice three months following consolidative radiotherapy completion and ten months following his initial presentation. His immediate cause of death was unknown.

## Discussion

This is the first report of which we are aware that describes a case of an isolated cardiac metastasis in a Caucasian patient with esophageal carcinoma in North America. This is likely due to the epidemiologic and geographic difference in rates of esophageal cancer, with the highest rates being in Eastern Asia and Eastern and Southern Africa and the lowest rates in Europe and North America [8].

The incidence of cardiac metastases and pericardial tumor extension found on autopsy in the general population has been reported to be 2.3% compared to 7.1% in patients with cancer [4]. According to autopsy studies, this is an increase and likely attributable to improved imaging modalities allowing for better detection and cancer care advancements that have extended the life expectancies of patients [4]. Cardiac metastases are more frequently observed in cases of primary lung cancer, breast cancer, hematologic malignancies, and melanoma [5]. Esophageal cancers are an uncommon cause of pericardial metastases, with isolated cardiac metastases being even more rare. A review by Bussani et al. revealed isolated cardiac metastases in 10 of 662 cancer cases, of which only one was due to esophageal carcinoma [1]. The clinical presentation of cardiac metastases varies depending on the site, size of the tumor, and extent of invasion. Arrhythmia, ischemic heart disease, and cardiomyopathy typically develop secondary to tumor invasion into the myocardium, whereas thromboembolism and obstruction of the cardiac tract are observed when the protruding tumor invades into the cardiac chamber. Cardiac tamponade can be seen with the pericardial invasion of the tumour, with heart failure in cases of extended infiltration. Symptoms tend to appear once the tumor reaches 4 cm in diameter [9].

Our patient presented with non-specific findings of syncope, intermittent melena, anemia, and unexplained weight loss, and was found to have poorly differentiated esophageal carcinoma confirmed on biopsy. A mildly enhancing lobulated circumscribed mass in the left atrium was incidentally identified on CT imaging. Similar to Oda et al., who highlighted the diagnostic challenges of evaluating cardiac metastases with cardiac MRI, our case showed discordance between cardiac MRI results that favored myxoma and F-18 FDG PET/CT findings, which showed an SUV_max_ value more typical of metastases [10,11]. The optimal imaging diagnostic workup for cardiac metastases remains an open question. In particular, F-18 FDG PET/CT is not yet included in the routine diagnosis of cardiac masses, although it may have substantial potential in the diagnostic algorithm and non-invasive preoperative determination of malignancy and metastatic spread of cardiac tumors [11]. It provides a functional assessment of the heart through SUV_max_ evaluation to delineate the malignant potential of non-specific masses and estimation of tumor invasion into cardiac layers and pericardium. It may also be useful for the evaluation of postoperative residual disease and early evaluation of response to chemotherapy. In our case, it showed intense FDG avidity at the cardiac mass with an SUV_max_ of 18, which is above the threshold for non-malignant masses and within the range for primary and secondary malignant cardiac tumors [11]. Given the presence of a single lesion in the left atrial wall, the mass was successfully resected for both diagnostic and therapeutic reasons. Pathological examination revealed a poorly differentiated carcinoma, identical to that of the esophageal mass. Interestingly, no lymphadenopathy or other potential distant metastases were identified.

The patient was initially treated with a single 8 Gy fraction of urgent hemostatic radiotherapy, and began palliative chemotherapy, bearing in mind the need for diligent monitoring given the risk of cardiac vasospasm from capecitabine and his recent cardiac surgery [9,12]. Another fraction of hemostatic radiotherapy was needed after he began anticoagulation for a pulmonary embolus, highlighting a common challenge for patients with in-situ upper abdominal primary cancers. In retrospect, a higher initial total dose of fractionated palliative radiotherapy may have prevented the need for retreatment; however, the hope was to control bleeding with as low a dose as necessary in order to minimize any radiotherapy-related toxicities that may have delayed the systemic therapy he needed. Unfortunately, systemic therapy was ultimately discontinued due to a decline in the patient’s performance status and ongoing weight loss. He received consolidative palliative radiotherapy (20 Gy in five fractions), after which he was able to eat solid foods without dysphagia and was devoid of pain and bleeding. His overall decline was likely due to a number of challenges such as blood loss, limited reserve, and poor appetite and energy following systemic therapy. Disease progression was unlikely to explain the decline given the stable imaging findings, and radiotherapy seemed to help his overall symptom burden. He ultimately died in hospice three months following completion of radiotherapy, which was ten months following his initial presentation.

## Conclusions

In summary, we present a unique case of a Caucasian male that contributes to the limited literature describing solitary cardiac metastases secondary to esophageal carcinoma. Given advances in imaging and other diagnostic techniques, we recommend an extensive workup of patients with suspected or possible isolated cardiac metastases in the setting of esophageal cancers to optimize management plans and prognosis estimates. Resection of solitary cardiac masses in this setting should be considered, when possible, for both diagnostic and therapeutic purposes. We believe that confirming metastatic disease in our patient spared him the potential toxicities of more aggressive curative-intent multimodality therapy and allowed him to set realistic goals during advance care planning. 
